# Frequency-Aware Degradation Modeling for Real-World Thermal Image Super-Resolution

**DOI:** 10.3390/e26030209

**Published:** 2024-02-27

**Authors:** Chao Qu, Xiaoyu Chen, Qihan Xu, Jing Han

**Affiliations:** Jiangsu Key Laboratory of Spectral Imaging and Intelligent Sense, Nanjing University of Science and Technology, Nanjing 210094, China

**Keywords:** real-world thermal image, super-resolution, degradation modeling, frequency-aware

## Abstract

The supervised super-resolution (SR) methods based on simple degradation assumptions (e.g., bicubic downsampling) have unsatisfactory generalization ability on real-world thermal images. To enhance the SR effect of real-world sceneries, we introduce an unsupervised SR framework for thermal images, incorporating degradation modeling and corresponding SR. Inspired by the physical prior that high frequency affects details and low frequency affects thermal contrast, we propose a frequency-aware degradation model, named TFADGAN. The model achieves image quality migration between thermal detectors of different resolutions by degrading different frequency components of the image from high-resolution (HR) to low-resolution (LR). Specifically, by adversarial learning with unpaired LR thermal images, the complex degradation processes of HR thermal images at low and high frequencies are modeled separately. Benefiting from the thermal characteristics mined from real-world images, the degraded images generated by TFADGAN are similar to LR thermal ones in terms of detail and contrast. Then, the SR model is trained based on the pseudo-paired data consisting of degraded images and HR images. Extensive experimental results demonstrate that the degraded images generated by TFADGAN provide reliable alternatives to real-world LR thermal images. In real-world thermal image experiments, the proposed SR framework can improve the peak signal-to-noise ratio (PSNR) and structural similarity degree (SSIM) by 1.28 dB and 0.02, respectively.

## 1. Introduction

Thermal detectors are passive sensors that capture the thermal radiation emitted by objects themselves in the range of the Long Wavelength Infrared (LWIR) spectrum. With the advantages of high penetration, high concealment, and all-weather observation, thermal detectors have a widespread and important application in military, medical, and pedestrian detection [1,2,3]. The spatial resolution of thermal images is generally low due to manufacturing technology constraints and the expensive cost of large sensor arrays, which limits the further exploitation of thermal detectors [4]. To reconstruct high-resolution (HR) images from low-resolution (LR) images, thermal image super-resolution (SR) algorithms that are practical and economical are of great research significance.

Numerous image SR methods have been proposed, including interpolation [5], sparse representation [6], and neighbor embedding [7]. In recent years, deep learning-based methods have made remarkable progress in thermal image SR tasks. The standard paradigm of deep learning is to learn the mapping from LR image to HR counterpart, this leads to a reliance on the large-scale dataset of real-world LR-HR image pairs. However, collecting real-world thermal LR-HR image pairs is extremely tedious and labor-intensive. Therefore, in most learning-based methods, the training data used consist of HR images from the real world, while LR images are artificially synthesized under simple degradation assumptions [8,9,10,11,12,13]. Although these SR methods achieve good reconstruction results on synthetic data, it is challenging to achieve satisfactory performance on real LR images. Specifically, this is because the degradation process of real-world HR thermal images is complex and unknown, involving factors such as blur, noise, contrast, etc. This is vastly different from the known simple degradation process (e.g., bicubic downsampling).

To address challenging real-world scenarios, several unsupervised SR schemes based on degradation modeling have been proposed [14,15,16,17]. These unsupervised methods utilize unpaired LR-HR datasets widely present in the real world to simulate degraded images, and then use the degraded images and their corresponding HR images to train the SR model. It is worth noting that, to ensure degradation quality, unpaired LR-HR datasets typically have scene similarity. By leveraging more realistic training data, deep SR networks have improved their ability to reconstruct images in real-world. In this process, the degradation model needs to maintain the similarity between the generated images and the HR images, and introduce the features of the unpaired LR images. Most degradation models are designed for visible images. They focus on detail degradation while strictly constraining the basic content of the degraded image to be consistent with the original image to prevent color shifts [15,17].

However, thermal detectors at different resolutions not only differ in thermal details but also vary significantly in thermal contrast. Therefore, it is difficult to accurately degrade thermal images solely based on degradation strategies for visible light images without considering the characteristics of thermal images. In addition, existing methods ignore the impacts of different frequency components on image degradation. The low-frequency components affect the image base content, such as contrast, contour, and structure, while the high-frequency components affect the image details. For this reason, the high-frequency and low-frequency components of the image are processed separately to achieve excellent reconstruction results in many image enhancement methods [18,19,20,21]. Similarly, contrast and noise are difficult to be handled separately when degrading images in the spatial domain, which may make degradation from HR to LR more challenging.

In this paper, we propose an unsupervised SR framework for thermal images, which improves the performance of SR models on real-world images through high-accuracy degradation modeling. Inspired by the physical prior that high frequency affects details and low frequency affects thermal contrast, we propose TFADGAN, a frequency-aware degradation model based on unpaired LR-HR thermal images. Specifically, the HR thermal image is decomposed into low-frequency components and high-frequency components before degradation. Through adversarial learning, the dual-frequency network implicitly models the degradation process from HR to LR for both high-frequency and low-frequency components, with the low-frequency network focusing on thermal contrast degradation and the high-frequency network focusing on detail degradation. To ensure degradation stability, we design a cross-frequency feature modulation (CFFM) module, embedded between the high-frequency network and the low-frequency network. This module utilizes low-frequency features to guide the encoding of high-frequency features, aligning the structural information between different frequency components. The degraded low-frequency and high-frequency components are inverted to obtain degraded image similar to the real LR image. The SR model establishes the mapping of degraded images to corresponding HR images to enhance generalization in the real world. In our framework, the degradation model and SR model are trained independently, and any SR network can be used in conjunction with the proposed degradation model.

In summary, the main contributions of this manuscript are as follows:

1. We propose a frequency-aware degradation model named TFADGAN based on unpaired unsupervised learning, which can generate realistic degraded images by transforming different frequency components of images from HR to LR.

2. We design a CFFM module in the dual-frequency degradation network, which utilizes low-frequency features to guide the encoding of high-frequency features, effectively suppressing artifact generation.

3. We conducted comprehensive experiments on both synthetic and real data. The results show that the degraded images generated by TFADGAN provide a reliable alternative to real-world LR images and significantly improve the performance of deep SR networks in the real world.

## 2. Related Work

### 2.1. Thermal Image Super-Resolution

In recent years, convolutional neural networks have demonstrated superior performance in visible image SR. When researchers extend the visible image SR reconstruction to thermal images, the first problem is the lack of high-quality thermal paired datasets. Inspired by the first image SR network SRCNN [22], Choi et al. proposed the thermal enhancement network, which has an overly simple structure and uses visible light image data for training [8]. He et al. used a cascaded CNN architecture and achieved a large-scale factor SR [9]. The DWCNN proposed by Gunnam Suryanarayana et al. is based on the multiscale saliency detection and the residuals learned by the deep CNN in the wavelet domain, which requires less computation while achieving excellent reconstruction quality [10]. Vishal Chudsama et al. proposed TherISuRNet, a thermal SR network based on an asymmetric progressive learning strategy, which enhances the reconstruction capability of deep models [11]. Chen et al. proposed IERN, a thermal SR network based on an iterative error reconstruction mechanism, with a satisfactory reconstruction of image details and edges [23]. The above studies improve the performance of thermal image SR networks. However, due to the use of artificially synthesized LR-HR data during the training process, it is challenging to generalize them effectively to real-world thermal images.

### 2.2. Unsupervised Image Super-Resolution

Although SR models have produced impressive results on synthetic datasets with known degradation assumptions, they tend to underperform in the real world. Unsupervised methods to solve the problem of lacking paired data are receiving increasing attention. UISR only relies on LR images for training and optimizes the details of SR images by maintaining the image style uniformity and mining the texture distribution characteristics in the image domain [24]. However, this method faces challenges in the restoration of images with severely damaged details. CinCGAN [25] and unsupervisedThSR [26] use the framework of CycleGAN [27] to learn the mapping of LR images to HR images using unpaired datasets. In these networks, cycle-consistency constraint is over-emphasized, resulting in inadequate training stability.

Bulat et al. [28], Chen et al. [14], FSSR [15], and DASR [17] focus on the image degradation process, learning the transformation from HR to LR. In the work of Bulat et al. [28] and Chen et al. [14], the degradation model and the SR model are trained jointly. The undesired results in the initial stage of the degradation training can negatively affect the SR training. In FSSR and DASR, the training of the degradation model and the SR model are separated, which can effectively circumvent the above problem. FSSR separates the high and low frequencies of the images, and then discriminates whether the high frequencies of the degraded images are similar to those of the real LR images. On this basis, DASR advocates domain-aware training to obtain superior SR results. However, since FSSR and DASR ignore the role of image low-frequency components in the degradation process, the generated degraded images still differ significantly from real LR images, resulting in unsatisfactory thermal image super-resolution performance.

## 3. Degradation Model

To obtain high-accuracy degraded images, it is necessary to understand how HR thermal images deteriorate into LR thermal images. From [29,30], it is known that the thermal imaging process experiences various kinds of blurring, such as optical blurring due to the diffraction limit of the imaging optical system and detector blurring due to the point spread function of the image detector. The optical and detector blurring process of the image can be expressed as:

(1) $$I_{hb}=I_{h}\times B,$$

where $I_{h}$ is the ideal HR thermal image with a size of m×n, $I_{hb}$ is the HR blurred thermal image, and *B* is the blur matrix. Secondly, during the thermal image sampling, the sensor size and transmission bandwidth will reduce the image resolution:

(2) $$I_{lb}=I_{hb}\times S,$$

where $I_{lb}$ is the LR blurred thermal image with a size of m/s×n/s and *S* is the downsampling matrix with a scale of *s*. In addition, the noise in the imaging process is not negligible. These noises can be divided into low-frequency noise and high-frequency noise, where the low-frequency noise is mainly 1/f noise and the high noise is mainly shot noise and thermal noise. In summary, the degenerate image $I_{l}^{dg}$ is expressed as

(3) $$I_{l}^{dg}=I_{lb}+N^{lf}+N^{hf}=I_{h}\times B\times S+N^{lf}+N^{hf},$$

where $N_{lf}$ is the low-frequency noise matrix, and $N_{hf}$ is the high-frequency noise matrix. Since it is difficult to acquire ideal HR thermal images without degradation in the real world, we use the thermal images captured by HR and high-quality thermal detectors instead. The thermal images captured by thermal detectors with different resolutions are different in not only the details related to high frequency, but also the thermal contrast related to low frequency. Therefore, for thermal images, the above degradation model can be optimized from the perspective of frequency domains. Before degradation, the original HR image is decomposed into low-frequency and high-frequency components:

(4) $$I_{h}^{lf}=I_{h}^{real}\times f_{l},\;I_{h}^{hf}=I_{h}^{real}\times f_{h},$$

where $I_{h}^{real}$ is the high-quality thermal image, $f_{l}$ and $f_{h}$ denote the low-pass filter and high-pass filter, respectively, and $I_{h}^{lf}$ and $I_{h}^{hf}$ denote the low-frequency component and the high-frequency component of the original image, respectively. The low-frequency and high-frequency of thermal images have different degradation processes:

(5) $$\begin{array}{cc} \bar{I_{l}^{lf}}= & D^{lf}\left(I_{h}^{lf}\right)=I_{h}^{lf}\times C\times S+N^{lf},\\ \bar{I_{h}^{hf}}= & D^{hf}\left(I_{h}^{hf}\right)=I_{h}^{hf}\times B\times S+N^{hf}, \end{array}$$

where *C* is the thermal contrast transformation matrix, $\bar{I_{h}^{lf}}$ and $\bar{I_{h}^{hf}}$ denote the degraded low-frequency component and high-frequency component, respectively, and $D^{lf}\left(\cdot \right)$ and $D^{hf}\left(\cdot \right)$ denote the low-frequency degradation function and high-frequency degradation function, respectively. Then, the low-frequency component and the high-frequency component are inverted to obtain the degraded image as follows:

(6) $$I_{l}^{dg}=\bar{I_{l}^{lf}}⊕\;\bar{I_{l}^{hf}},$$

where ⊕ denotes the inversion process. It is worth noting that neither the image frequency decomposition nor its inversion introduces additional information loss. Because the degradation in the real world is unknown, we adopt an implicit modeling approach to learn the transformation from HR images to LR images. This process does not require explicit parameterized representations of blur matrix B, downsampling matrix S and contrast matrix *C*. Additionally, the low-frequency degradation function $D^{lf}\left(\cdot \right)$ and the high-frequency degradation function $D^{hf}\left(\cdot \right)$ is the key to the degradation process. In our method, the low-frequency degradation function $D^{lf}\left(\cdot \right)$ and the high-frequency degradation function $D^{hf}\left(\cdot \right)$ of HR thermal images are fitted by different network branches through adversarial learning with unpaired LR thermal images.

## 4. Proposed Method

**Figure 1:**
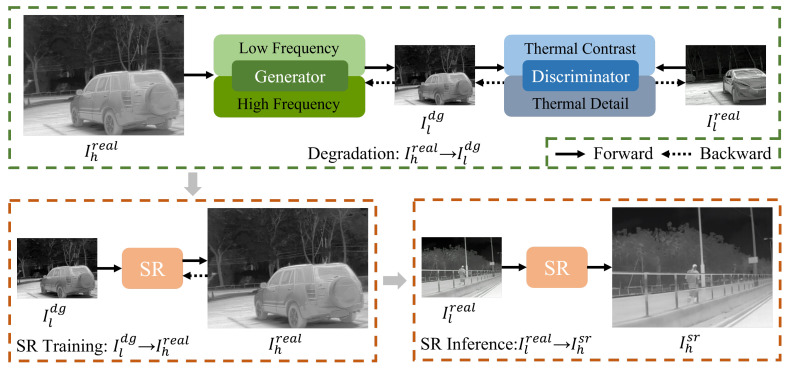
The overview of our proposed framework. The green box represents the degradation process, and the orange box represents the super-resolution process. In the degradation process, real HR thermal image $I_{h}^{real}$ is transformed into degraded image $I_{l}^{dg}$ through adversarial learning using unpaired data captured by detectors with different resolutions. The SR process is divided into training and inference stages. During the SR training stage, the SR model is trained based on pseudo-paired data composed of LR degraded image $I_{l}^{dg}$ and real HR thermal image $I_{h}^{real}$. In the SR inference stage, real LR thermal image $I_{l}^{real}$ is reconstructed into HR image by the SR model.

Based on the degradation model in Section 2, we propose an SR framework for unpaired thermal images based on frequency-aware degradation. Figure 1 illustrates the framework, which consists of a degradation process and an SR process. Given a real-world HR thermal image $I_{h}^{real}$ and an unpaired LR thermal image $I_{l}^{real}$, the degraded image of $I_{h}^{real}$ is denoted as $I_{l}^{dg}$ and the upgraded image of $I_{l}^{real}$ is denoted as $I_{h}^{sr}$. In the degradation process, the proposed TFADGAN implicitly models the complex degradation process from $I_{h}^{real}$ to unpaired $I_{l}^{real}$, generating degraded images $I_{l}^{dg}$. In the SR training phase, the SR model is trained using pseudo-paired data composed of degraded image $I_{l}^{dg}$ and real HR thermal image $I_{h}^{real}$. In the SR inference phase, the SR model reconstructs the HR thermal image $I_{h}^{sr}$ from the real LR thermal image $I_{l}^{real}$.

### 4.1. Thermal Image Frequency-Aware Degradation

**Figure 2:**
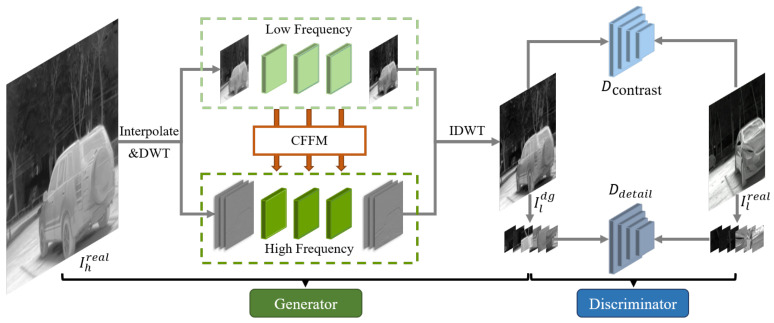
The structure of the proposed degradation model TFADGAN. The model consists of a generator, a style discriminator and a texture discriminator. The generator uses a two-branch network in which the cross-frequency feature modulation module is bridged between the different branches.

In this section, the thermal image frequency-aware degradation model (TFADGAN) is introduced. The TFADGAN consists of a dual-frequency degradation generator and a dual-discriminator. The structure of TFADGAN is depicted in Figure 2. The real HR image $I_{h}^{real}$ is downsampled to the same size as the real LR image $I_{l}^{real}$. Then the dual-frequency degradation generator degrades the downsampled image at high and low frequencies. With the thermal detail discriminator and the thermal contrast discriminator, the high-accuracy degraded image $I_{l}^{dg}$ is obtained.

#### 4.1.1. Dual-Frequency Decomposition

According to Equation (4), we use the discrete wavelet transform (DWT) to decompose the original image into low-frequency sub-bands and high-frequency sub-bands. Due to the double orthogonal property of inverse DWT (IWDT), the original image can be accurately reconstructed using the sub-band images even though their resolution is half that of the original image. Because of the computational simplicity and powerful multi-frequency information characterization of Haar wavelets [31], we use Haar wavelets as the basis functions of the DWT. Its low-pass filter $f_{l}$ and high-pass filter $f_{h}$ are defined as

(7) $$f_{l}=\frac{1}{\sqrt{2}}{\left[1,1\right]}^{T},f_{h}=\frac{1}{\sqrt{2}}{\left[1,-1\right]}^{T}.$$

The cutoff frequencies of both $f_{l}$ and $f_{h}$ are half of the Nyquist frequency. In first-level of the wavelet transform, the original image *I* is filtered with low-pass and high-pass and downsampled along the row and column directions, respectively. After transform, four sub-band images $I_{LL}$, $I_{LH}$, $I_{HL}$, and $I_{HH}$ are obtained. the first sub-band $I_{LL}$ corresponds to the low-frequency information of the original image *I*, and the remaining sub-bands $I_{LH}$, $I_{HL}$, and $I_{HH}$ correspond to the high-frequency contents in the horizontal, vertical, and diagonal directions, respectively. The low-frequency sub-band $I_{h}^{lf}$ and high-frequency sub-band $I_{h}^{hf}$ corresponding to a high-resolution thermal image $I_{h}^{real}$ are as follows:

(8) $$I_{h}^{lf}=\left\{I_{LL}\right\},I_{h}^{hf}=\left\{I_{LH},I_{HL},I_{HH}\right\}.$$

#### 4.1.2. Cross-Frequency Feature Modulation

The degradation of low- and high-frequency sub-bands using different network branches faces a huge challenge. Because the low-frequency branch focuses on the thermal contrast, and the high-frequency branch focuses on the image details, different branches may produce mismatched content at corresponding positions. In the absence of any guiding measures between the high-frequency branch and the low-frequency branch, artifacts and structural distortion may occur in degraded images. Compared to the high-frequency sub-bands, the low-frequency sub-bands have larger modal values and contain rich structural information. The study demonstrates that the high-frequency sub-band enhancement based on the regional characteristics of the low-frequency sub-band can obtain a high-quality thermal reconstruction image [19]. Inspired by the above physical properties and SEAN [32], we designed the cross-frequency feature modulation (CFFM) module, which ensures the consistency of structural information in the low and high frequencies of the image and makes the degradation results more realistic and natural.

**Figure 3:**
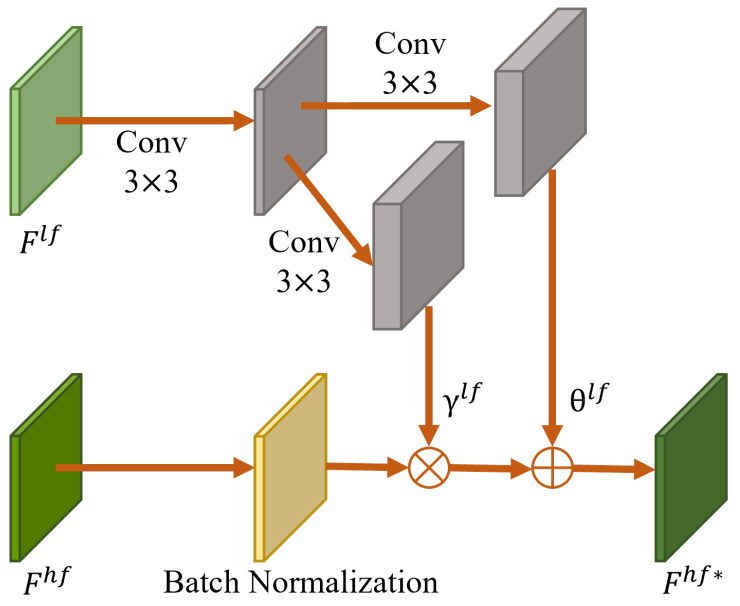
The structure of the cross-frequency feature modulation (CFFM) module. The $\gamma^{lf}$ and $\theta^{lf}$ are the modulation parameters to be learned.

Specifically, CFFM module utilizes the modulation parameters learned from low-frequency features to adjust high-frequency features in the channel dimension in order to align low-frequency features with high-frequency features. As shown in Figure 3, high-frequency features are first normalized since the mean and variance of convolutional features have a significant influence on the style of images [33]. Then, the scale parameter $\gamma^{lf}$ and the bias parameter $\theta^{lf}$ learned from the low-frequency features $F^{lf}=\left\{F_{1}^{lf},\ldots ,F_{n}^{lf}\right\}$ are injected into the high-frequency features $F^{hf}=\left\{F_{1}^{hf},\ldots ,F_{n}^{hf}\right\}$. The modulated high-frequency features $F^{hf*}$ are defined as

(9) $$F^{hf*}=\gamma^{lf}\frac{F^{hf}-\mu^{hf}}{\sigma^{hf}}+\theta^{lf},$$

where $\mu^{hf}$ and $\sigma^{hf}$ are the mean and standard deviation of the high-frequency features $F^{hf}$ along the channel dimension, and $\gamma^{lf}$ and $\theta^{lf}$ are the scale parameters and deviation parameters that can be updated.

#### 4.1.3. Dual-Frequency Degradation Generator

**Figure 4:**
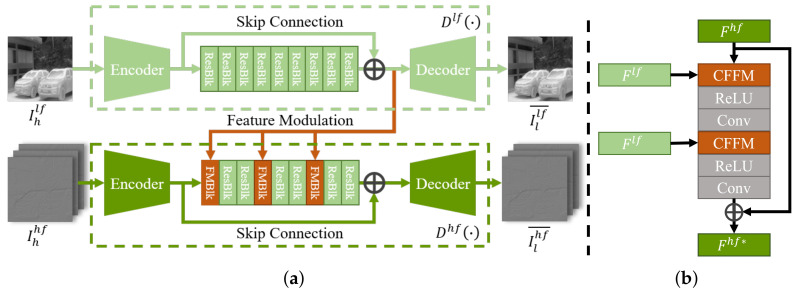
(**a**) The structure of the generator of TFADGAN. The generator consists of a low-frequency branch and a high-frequency branch. The low-frequency sub-bands and high-frequency sub-bands are degenerated through the low-frequency branch and the high-frequency branch, respectively. During degradation, the low-frequency features modulate the high-frequency features at three different depths of the network through the FMblk. (**b**) The structure of FMBlk.

The dual-frequency degradation generator consists of two different branches for calculating the low-frequency degradation function $D^{lf}\left(\cdot \right)$ and the high-frequency degradation function $D^{hf}\left(\cdot \right)$ in Equation (5), respectively. Figure 4 illustrates the structure of the generator. The low-frequency branch is composed of an encoder, 9 ResNet blocks [34], and a decoder. Each ResNet block (ResBlk) contains two convolution layers (kernel size 3 × 3, number of channels 64) and a ReLU activation in the middle. Similar to SEAN, we modify the ResBlk to obtain the FMBlk. As illustrated in Figure 4, the FMBlk consists of two convolution layers whose scales and biases are modulated by the CFFM module, respectively. The high-frequency degradation branch has a similar structure to the low-frequency branch, except that three ResBlks are replaced by FMBlks.

The low-frequency sub-bands $I_{h}^{lf}$ and high-frequency sub-band $I_{h}^{lf}$ of the input image are treated to obtain the corresponding degraded sub-bands $\bar{I_{l}^{lf}}$ and $\bar{I_{l}^{hf}}$, respectively. While the low-frequency branch completes degradation learning, the low-frequency features output from the last ResBlk are injected into the high-frequency branch via FMBlk. The encoding of high-frequency features is modulated by low-frequency features, which effectively improves the structural consistency between high-frequency information and low-frequency information. Then the two ResBlks further extract the features of the modulated high-frequency information. After three times of feature modulation, the degraded sub-bands $\bar{I_{l}^{lf}}$ and $\bar{I_{l}^{hf}}$ generate the degraded image by IWDT.

#### 4.1.4. Discriminator for Detail and Contrast

Double discriminators are designed, including a thermal contrast discriminator $D_{contrast}$ and a detail discriminator $D_{detail}$, to increase the similarity between the degraded image and the real image in blur, noise and thermal contrast. The global-scale discrimination is crucial to adjust the image contrast, and local-scale discrimination can effectively promote the degradation of detail texture. Therefore, the $D_{contrast}$ and the $D_{detail}$ jointly constrain the degraded image from the global scale and the local scale, respectively. Among them, the $D_{detail}$ discriminates six 48 × 48 image blocks randomly cropped from the degraded image and the real LR image, respectively. Both the thermal contrast discriminator $D_{contrast}$ and the detail discriminator $D_{detail}$ use the relativistic average discriminator [35]. The standard function of the relativistic average discriminator is:

(10) $$\begin{array}{c} D_{Ra}\left(I_{l}^{real},I_{l}^{dg}\right)=\sigma \left(C\left(I_{l}^{real}\right)-E\left[C(I_{l}^{dg})\right]\right),\\ D_{Ra}\left(I_{l}^{dg},I_{l}^{real}\right)=\sigma \left(C\left(I_{l}^{dg}\right)-E\left[C(I_{l}^{real})\right]\right), \end{array}$$

where $I_{l}^{real}$ is the real LR thermal image, $I_{l}^{dg}$ is the degraded image, $D_{Ra}\left(\cdot \right)$ represents the final output of the discriminator, C(·) represents the discriminator network, E[·] represents the mean calculation, and σ represents the sigmoid function.

#### 4.1.5. Loss Functions

We combine multiple loss functions to train the image degradation model. To keep the low-frequency sub-band of the images similar before and after degradation, we use low-frequency loss $L_{lf}$ to constrain the distance between the degraded low-frequency sub-band $\bar{I_{l}^{lf}}$ and the downsampled low-frequency sub-band $I_{h}^{lf}$:

(11) $$\begin{array}{cc} L_{lf}= & \frac{1}{N}\sum_{i=1}^{N}\left|\right|{\left(\bar{I_{l}^{lf}}\right)}_{i}-{\left(I_{h}^{lf}\right)}_{i}{\left|\right|}_{1}\\ +1- & \frac{1}{N}\sum_{i=1}^{N}SSIM\left({\left(\bar{I_{l}^{lf}}\right)}_{i},{\left(I_{h}^{lf}\right)}_{i}\right), \end{array}$$

where $\left|\right|\cdot {\left|\right|}_{1}$, ${\left(\bar{I_{l}^{lf}}\right)}_{i}$ and ${\left(I_{h}^{lf}\right)}_{i}$ represent $l_{1}$ norm, i−th low-frequency sub-bands of degraded image and downsampled image, respectively. The SSIM(·) is the structural similarity index (see [36] for more details). Moreover, we use the Total-Variation (TV) loss [37] to help the degradation effect to be more natural. The TV loss is presented as

(12) $$\begin{array}{c} L_{tv}=\frac{1}{N}\sum_{i=1}^{N}\left(|\right|\nabla_{h}{\left(I_{l}^{dg}\right)}_{i}{\left|\right|}_{2}+\left|\right|\nabla_{w}{\left(l_{l}^{dg}\right)}_{i}{\left|\right|}_{2}), \end{array}$$

where $\left|\right|\cdot {\left|\right|}_{2}$ represents $l_{2}$ norm. The $\nabla_{h}$ and $\nabla_{w}$ are functions that compute the horizontal and vertical gradients of $I_{l}^{dg}$. The total loss is defined as

(13) $$\begin{array}{cc} L_{total} & ={\lambda_{1}L}_{lf}+\lambda_{2}L_{tv}+\lambda_{3}L_{adv}, \end{array}$$

where $\lambda_{1}$, $\lambda_{2}$ and $\lambda_{3}$ represent the weights of each loss, which are set to $1\times 10^{-2}$, $1\times 10^{-3}$ and $1\times 10^{-2}$, respectively. The $L_{adv}$ terms contains both detail and contrast discriminator losses.

### 4.2. Degradation-Based Super-Resolution

The SR network is trained based on the degraded images generated by TFADGAN. Compared to the joint training method of degradation and SR, our framework is trained in two steps, which can minimize the disturbance to SR caused by the bad results in the initial stage of degradation. Furthermore, our framework is flexible enough that any supervised SR model can be paired with the degradation model TFADGAN to achieve different SR effects.

In this paper, we choose PixelSR for pixel loss and PerceSR for perceptual quality to experiment. The PixelSR is exemplified by TherISuRNet [11], which uses a progressive upscaling strategy based on asymmetric residual learning to achieve efficient SR reconstruction of thermal images. PerceSR is exemplified by ESRGAN [38], and its generator uses the Residual-in-Residual Dense Block to extract image features and employs the relative loss function from the Relativistic GAN to effectively improve the visual quality.

### 4.3. The Algorithm Combining Degradation and Super-Resolution

In order to show our proposed algorithm more intuitively, Algorithm 1 shows the whole process of combining the TFADGAN and super-resolution method.

The two main processes described in each line are as follows:

- Lines 1–5: The degradation model TFADGAN is trained using unpaired LR-HR thermal images. Through *N* iterations, TFADGAN generates the reliable degraded LR thermal image $I_{l}^{dg}$.
- Lines 6–10: The SR network is trained using degraded LR thermal image $I_{l}^{dg}$ and corresponding HR thermal image $I_{h}^{real}$. Through *M* iterations, the SR network reconstructs high-quality HR thermal image $I_{h}^{sr}$.

**Algorithm 1** Complete algorithm with TFADGAN and SR method**Input:** HR thermal image $I_{h}^{real}$, Unpaired LR thermal image $I_{l}^{real}$
**Output:** Degraded LR thermal image $I_{l}^{dg}$, Reconstructed HR thermal image $I_{h}^{sr}$

 1: **for** *i* in range(*N*) **do**

 2:    Select unpaired HR thermal image $I_{h}^{real}$ and LR thermal image $I_{l}^{real}$

 3:    Train TFADGAN to generate degraded LR thermal image $I_{l}^{dg}$ corresponding to HR thermal image $I_{h}^{real}$ and compute total loss in Equation (13)

 4:    Update TFADGAN

 5: **end for**

 6: **for** *i* in range(*M*) **do**

 7:    Select the degraded LR thermal image $I_{l}^{dg}$ and paired HR thermal image $I_{h}^{real}$

 8:    Train SR network, generate reconstructed HR thermal image $I_{h}^{sr}$ and compute reconstruction loss

 9:    Update SR network

10: **end for**


## 5. Experiment and Results

### 5.1. Datasets and Details

*(1) Synthetic Datasets:* An open dataset [39], namely CVC-09, is used to create synthetic thermal images. We randomly select 2000 thermal images from CVC-09, 1000 of which were used as HR images with a resolution of 640 × 480, and the other 1000 were manually degraded to obtain LR images with a resolution of 320 × 240. Then we obtain 1000 unpaired LR-HR images as the training set. The degradation is performed as follows: first, the HR images are downsampled using bicubic interpolation with a factor of 2, and then Gaussian noise (with mean value 0 and standard deviation 10) is added to the downsampled images. In addition, 100 images are selected and degraded in the same way to obtain LR images as the test set.

*(2) Real-world Datasets:* The open thermal image dataset provided by Rivadeneira is used as the real-world dataset [40]. This dataset consists of images captured by detectors of different resolutions, with each resolution containing 951 training images and 50 test images. Detectors of different resolutions exhibit differences in noise, blur, and thermal contrast, and each degradation factor is unknown. It is worth noting that the different resolution images are not aligned to each other by pixels. In this work, we use 320 × 240 resolution images as LR images and 640 × 480 resolution images as HR images.

*(3) Train Details:* In the unsupervised image degradation stage, the same training scheme is used for the synthetic and real-world datasets. We do random cropping and random horizontal flipping to obtain 128 × 128 patches. In the network, we use Adam optimizer, set batch size to 4, epoch to 200, and initial learning rate to $1\times 10^{-4}$. In the supervised super-resolution stage, both PixelSR and PerceSR follow the default parameter settings of the corresponding network. The scale factor of SR networks is set to 2. We use NVIDIA TITAN RTX to implement the above training process.

### 5.2. Evaluation Metrics

To quantitatively compare the performance of different SR methods for thermal images, we use several reference image quality metrics. For example, the Peak Signal-to-Noise Ratio (PSNR) and Structural Similarity Index Measure (SSIM) are classical methods used for image distortion measurements. Since the LR images and HR images in the dataset [40] provided by Rivadeneira et al. are not fully pixel-aligned, it is inaccurate directly use SR images and HR images to calculate the reference evaluation index for assessing image qualities. Therefore, it is necessary to align the SR images and HR images before evaluating the images. We choose the SIFT [41] operator to obtain the feature key points between the SR images and the HR images and then align the two images based on these feature points. To remove the influence of the black area around the aligned image on the image evaluation, only the center cropped area (50%) of images is evaluated.

In addition, we used the image contrast function [42] to calculate the deviation of gray levels. The contrast distortion of HR and SR is calculated as follows:

(14) $$\begin{array}{c} C\left(I\right)=\frac{1}{hw}\sum_{x=1}^{w}\sum_{y=1}^{h}I^{2}\left(x,y\right)-|\frac{1}{hw}\sum_{x=1}^{x}\sum_{y=1}^{h}I(x,y){|}^{2},\\ Contrast=10*\left|\log_{10}C(I_{SR})-\log_{10}C(I_{HR})\right|, \end{array}$$

where *w* is the width of the image and *h* is the height of the image, I(x,y) is the gray level of the pixel at (x,y). Among the above evaluation metrics, higher PSNR and SSIM values indicate less distortion of SR image; less contrast distortion means that the contrast between SR image and HR image is closer.

### 5.3. Ablation Study

In this section, we perform ablation experiments to investigate the contributions of different components in the degradation model TFADGAN, including frequency decomposition (FD), CFFM module, thermal contrast discriminator, thermal detail discriminator, and loss function. In these experiments, we choose PixelSR [11] as the SR network.

*(1) Frequency decomposition:* The wavelet transform is used to separate the high and low frequencies of an image. The high and low frequencies are degraded separately and finely by a two-branch network. To demonstrate the effectiveness of frequency decomposition, we do not use frequency decomposition and use single-branch network instead. As presented in the Table 1, without frequency decomposition, the PSNR and SSIM values of SR decrease. This is because the degradation ability of the degradation network in each frequency band decreases after removing the frequency decomposition, which leads to the poor performance of SR network.

*(2) CFFM module:* The CFFM module is used to keep the degraded image high-frequency features matched with the low-frequency characteristics. To demonstrate the effectiveness of the CFFM module, we removed this module and retrained the degraded and SR network. From Table 1, it can be found that SR network performs poorly in all metrics. Because the high and low frequencies are degraded along their respective directions during degraded image generation, producing structural inconsistencies between each other.

*(3) Discriminator for contrast and detail:* The detail discriminator is used to reduce the texture differences between the degraded image and the real image. The contrast discriminator is used to reduce the style difference between the degraded image and the real image. As shown in Table 1, the absence of contrast discriminator and detail discriminator in TFADGAN both weaken the performance of SR network.

*(4) Loss Function:* We quantitatively measure the contribution of each term in the loss function in Equation (13) by iteratively adding each component and retraining the network from scratch. The performance evaluation was conducted on the real dataset, and the results were summarized in the table. As shown in Table 2, introducing the low-frequency loss $L_{lf}$ and the TV loss $L_{tv}$ significantly boost PSNR by 1.00 dB and 0.16 dB, respectively.

### 5.4. Comparison with State-of-the-Art Methods

We compare our method with several competitive unsupervised image SR methods, including Bulat et al. [28], FSSR [15], DASR [17], and Rivadeneira et al. [26]. Among them, Bulat et al. and Rivadeneira et al. are for collaborative training, and FSSR and DASR are for two-step training. The code of Rivadeneira et al. is implemented by ourselves, and Bulat et al., FSSR, and DASR are provided by the authors. In our framework, the combination of the degraded network TFADGAN and the SR network PerceSR is named TFADGAN-PerceSR, and the combination of the degraded network TFADGAN and the SR network PixelSR is named TFADGAN-PixelSR.

*(1) Comparisons on Synthetic Datasets:* Table 3 shows the test results of different unsupervised SR methods on the synthetic dataset. Our methods TFADGAN-PercepSR and TFADGAN-PixelSR achieved all suboptimal and optimal results on the average PSNR and SSIM. On the synthetic dataset, TFADGAN-PixelSR outperforms other unsupervised super-resolution methods by at least 1.57 dB on the average PSNR and has a significant advantage in contrast distortion.

**Figure 5:**
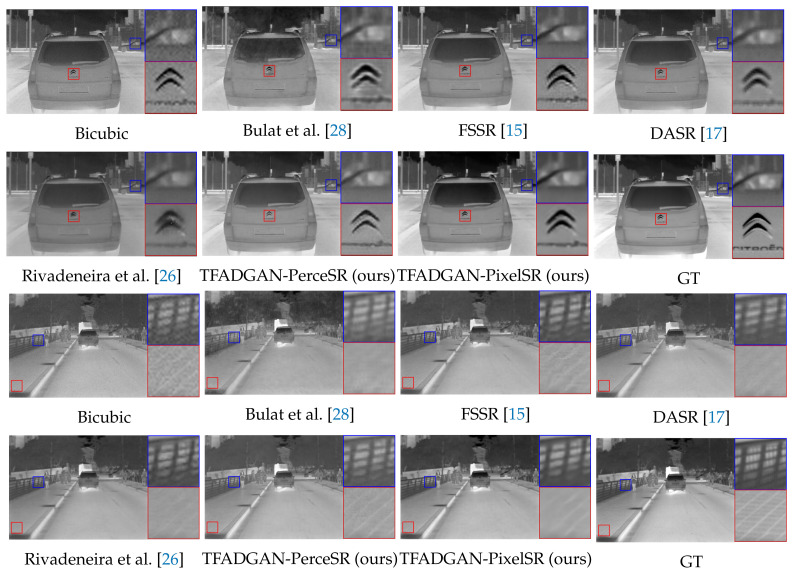
Visual comparison between our model and the state-of-the-art unsupervised methods on the synthetic dataset with scale ×2.

Figure 5 shows the SR results on part of the test set on the synthetic data. Bulat et al. amplify the noise while reconstructing the LR image, and its SR result on the synthetic image is not satisfactory. The SR results of FSSR and DASR showed different degrees of blurring because the degradation process ignored the role of low-frequency information in the images. Rivadeneira et al. utilizes the structure of CycleGAN [27] to facilitate image style transformation but is inefficient in recovering image detail. Compared with other methods, TFADGAN-PerceSR has a superior texture recovery ability and TFADGAN-PixelSR can maintain the natural image perception while suppressing noise.

*(2) Comparisons to Real-world Datasets:* Table 4 shows the test results of multiple SR methods on the real-world dataset. Our TFADGAN-PercepSR and TFADGAN-PixelSR achieved all optimal and suboptimal scores on real-world data for the three metrics. As can be observed from the table, our framework is highly competitive.

**Figure 6:**
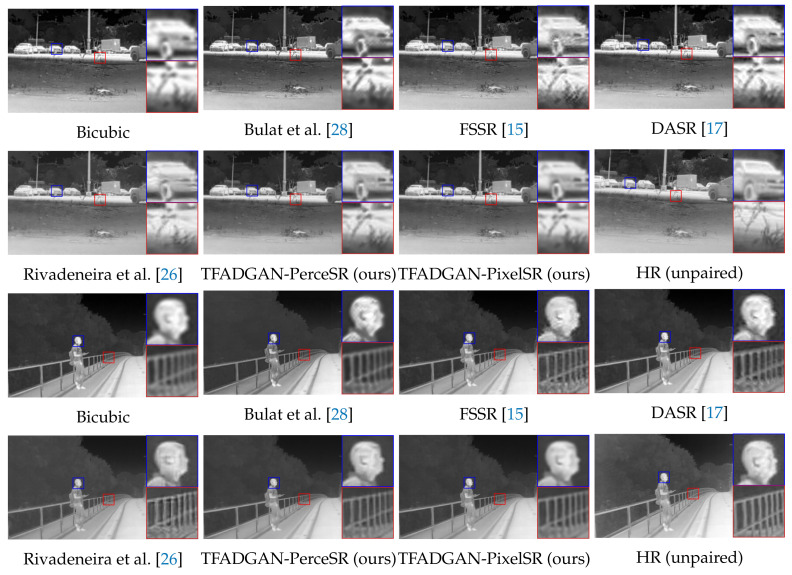
Visual comparison between our model and the state-of-the-art unsupervised methods on the real-world dataset with scale ×2.

Figure 6 illustrates SR results of different models. Since the degradation model and the SR model are jointly trained in Bulat et al., the undesirable effects of degradation induce SR to produce artifacts. There is more noise and blurred object edges in the SR images of FSSR and DASR. These unsatisfactory results may be attributed to the lack of appropriate guidance of high-frequency and low-frequency information in degradation procedures. The SR results of Rivadeneira et al. have a distortion in detail recovery, such as the car headlight in Figure 8a. Based on the proposed unsupervised SR network framework, both TFADGAN-PerceSR and TFADGAN-PixelSR exhibit excellent SR ability for real LR thermal images. TFADGAN-PerceSR oriented in the perceptual quality direction tends to generate SR images with clear textures and details. SR results of TFADGAN-PixelSR with pixel-wise supervision have less image noise.

### 5.5. Effectiveness of TFADGAN

We demonstrate the effectiveness of TFADGAN from both the degradation and SR perspectives on the real-world datasets. The effects of degradation methods were compared, including bicubic interpolation, FSSR-DSGAN in FSSR [15], DASR-DSN in DASR [17], and TFADGAN. Additionally, we evaluate the impact of different degradation models on SR networks. The PerceSR [11], an SR network oriented to the perceptual quality direction, and the PixelSR [38], an SR network oriented to the pixel loss direction, are used for image reconstruction with the scale factor set to 2.

**Figure 7:**
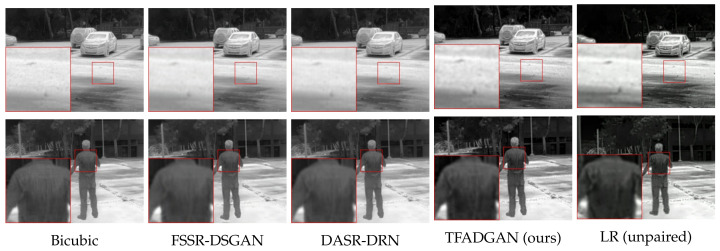
Visual comparison between TFADGAN and other degradation models on the real-world dataset with downsampling scale ×2.

The degraded images of each method and the corresponding real LR images are presented in Figure 7. Compared with bicubic interpolation, FSSR-DSGAN, and DASR-DSN, the degraded images generated by TFADGAN have the speckled background and blurred targets closer to the real LR images. In addition, the degraded TFADGAN images have a similar contrast to the real LR images as a result of the low-frequency network branch and contrast discriminator for style transformation.

**Figure 8:**
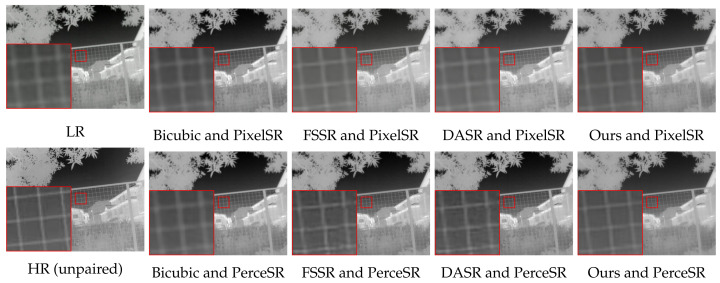
Comparison of SR results of different combinations of degradation models and SR models.

The results of the combinations formed by different degradation models and SR models are shown in Table 5. With the same super-resolution network, the paired data generated by TFADGAN can significantly improve the PSNR and SSIM values of SR results and effectively reduce the contrast distortion. When using the PerceSR as a SR network, our framework can improve PSNR and SSIM by 1.28 dB and 0.02, respectively. The different SR results are illustrated in Figure 8. When the degradation model is TFADGAN, the images reconstructed by the SR network are clear and have a natural visual appearance. When the degradation model is other methods, the images reconstructed by the SR network appear blurred, noisy or contrast-biased.

The above experiments demonstrate that the degraded images generated by TFADGAN are more similar to the real LR images. Compared with other degradation methods, the LR-HR paired data generated by TFADGAN can stimulate the performance of SR networks and effectively improve the image quality of SR results.

### 5.6. Model Complexity Analysis

The proposed framework consists of the degradation process and the SR process. The degradation model TFADGAN simulates the process from HR to LR, providing more realistic data for the subsequent training of the SR model. Therefore, the degradation model TFADGAN does not increase the computational cost and complexity of the SR model during the inference process.

To thoroughly analyze the balance between the effectiveness and efficiency of the degradation models, we investigate the complexity of different models. Specifically, we provide the model size and the number of multiply-accumulate operations (MACs) to measure the efficiency of models. Table 6 lists the network parameters and the number of MACs of FSSR-DSGAN in FSSR [15], DASR-DSN in DASR [17], and TFADGAN. Although TFADGAN does not have a significant advantage in model size compared to other methods, it has only 8.41G MACs due to its efficient structural design.

## 6. Limitations and Prospects

The proposed degradation model TFADGAN utilizes unpaired data to build the transformation from real-world HR images to LR images. This method effectively reduces the difficulty of collecting real-world paired LR-HR datasets. However, if there is a significant domain difference between the application scenario of the degradation model and the training data, it may lead to unstable degradation effects. Augmenting proprietary scene data and fine-tuning the model may help alleviate this issue.

Furthermore, we observe that TFADGAN also performs well in noise modeling. Based on the proposed unsupervised framework, TFADGAN has promising applicability in other low-level image tasks. In the future, we plan to extend this effective degradation method to other spectral images to solve the SR and denoising problems.

## 7. Conclusions

We use a novel training paradigm to address the problem of the limited real-world generalization of thermal images SR based on simple degradation assumptions. We design a frequency-aware degradation model for thermal image, named TFADGAN, which can be used in combination with any supervised SR network. Extensive experiments demonstrate that TFADGAN simulates a complex degradation process closer to the real world through an unsupervised approach. The degraded images generated by TFADGAN provide a reliable alternative to LR images. TFADGAN can sufficiently motivate the performance of SR networks and make SR networks generalize well on real-world thermal images.

## Figures and Tables

**Table 1:** Ablation study on the effectiveness of frequency decomposition (FD), CFFM module, contrast discriminator, and detail discriminator in the TFADGAN. The best and second-best results are shown in red and blue.

Degradation	PSNR	SSIM	Contrast
TFADGAN *w/o* FD	24.13	0.7695	0.6808
TFADGAN *w/o* CFFM	23.09	0.7560	0.8877
TFADGAN *w/o* $D_{contrast}$	23.91	0.7680	0.6696
TFADGAN *w/o* $D_{detail}$	24.09	0.7727	0.6244
TFADGAN	24.27	0.7758	0.6337

**Table 2:** Ablation study on the loss function design. The best results are shown in red.

$L_{adv}$	$L_{lf}$	$L_{tv}$	PSNR	SSIM	Contrast
✓			23.27	0.7576	1.0844
✓	✓		24.11	0.7723	0.7359
✓	✓	✓	24.27	0.7758	0.6337

**Table 3:** Comparison with unsupervised SISR methods on the synthetic dataset. The best and second best results are shown in red and blue.

Method	PSNR	SSIM	Contrast
Bicubic	27.95	0.7659	1.6067
Bulat et al. [28]	26.01	0.8445	1.9850
FSSR	28.71	0.8833	1.6955
DASR	28.59	0.8926	1.7350
Rivadeneira et al. [26]	28.21	0.8913	2.2993
TFADGAN-PerceSR (ours)	29.37	0.8941	1.9053
TFADGAN-PixelSR (ours)	30.28	0.9304	0.4465

**Table 4:** Comparison with unsupervised SISR methods on the real-world dataset. The best and second best results are shown in red and blue.

Method	PSNR	SSIM	Contrast
Bicubic	22.95	0.7431	1.0783
Bulat et al. [28]	20.58	0.7332	0.7434
FSSR	22.75	0.7237	1.0175
DASR	22.73	0.7264	1.0660
Rivadeneira et al. [26]	23.43	0.7513	0.8068
TFADGAN-PerceSR (ours)	24.17	0.7610	0.6522
TFADGAN-PixelSR (ours)	24.27	0.7758	0.6337

**Table 5:** Test set results of different combinations of degradation models and super-resolution models. The best results are shown in red.

Degradation	SR	PSNR	SSIM	Contrast
Bicubic	PixelSR	22.99	0.7550	0.9998
FSSR-DSGAN		23.50	0.7731	0.6115
DASR-DSN		23.30	0.7715	0.6195
TFADGAN (ours)		24.27	0.7758	0.6337
Bicubic	PerceSR	23.35	0.7557	0.9330
FSSR-DSGAN		23.32	0.7406	0.8301
DASR-DSN		23.07	0.7344	0.9990
TFADGAN (ours)		24.17	0.7610	0.6522

**Table 6:** Network parameters and MACs of the degradation models. MACs are calculated on 128 × 128 image patches.

Method	FSSR-DSGAN	DASR-DSN	TFADGAN
Parameters (M)	0.59	0.63	28.06
MACs (G)	9.69	9.83	8.41

## Data Availability

Data are contained within the article.
